# Ejercicio físico en el dolor musculoesquelético en tiempos de confinamiento social por Covid 19

**DOI:** 10.15649/cuidarte.2115

**Published:** 2021-09-10

**Authors:** Cristhian Santiago Bazán

**Affiliations:** 1 Tecnólogo Médico en Terapia física y rehabilitación, Doctor en educación, Centro de Rehabilitación Integral Física Funcional. Lima, Perú. Email: cristhiansantiagob@gmail.com Autor de correspondencia Centro de Rehabilitación Integral Física Funcional Perú cristhiansantiagob@gmail.com

Estimada editora,

El COVID-19 es una enfermedad causada por el virus del Síndrome Respiratorio Agudo Severo Coronavirus 2 (SARS-CoV-2), desarrollando una emergencia de salud pública en diferentes países a nivel mundial(1).

La pandemia COVID-19 ha obligado a las autoridades públicas sanitarias a nivel mundial a imponer un confinamiento social obligatorio como estrategia de contención epidemiológica. El confinamiento social tuvo un impacto negativo en los niveles de actividad física, la calidad del sueño y el bienestar en un grupo de adultos físicamente activos. Las autoridades de salud pública deben ser conscientes de que las personas que suelen llevar un estilo de vida activo, podrían ser particularmente susceptibles a tales trastornos(2). Así mismo la pandemia Covid 19 afectado los niveles de actividad física a consecuencia del confinamiento social obligatorio siendo este de suma importancia en la prevención de diferentes problemas de la salud.

Por otro lado, el brote del COVID-19 a nivel mundial ha desencadenado una pandemia que pone en peligro la salud mundial. El mundo del deporte también está sufriendo enormes consecuencias, como la suspensión de los Juegos Olímpicos de Tokio 2020(3) y otras disciplinas de distintas federaciones del deporte a nivel mundial.

Los factores psicológicos relacionados con el COVID-19 pueden exacerbar la experiencia del dolor y complicar el tratamiento del dolor por el impacto psicológico de COVID-19(4). Así mismo los pacientes con que padecer un transtorno muscular esquelético crónico reportaron un mayor nivel de estrés autoevaluado, que se correlacionó con un aumento significativo de la intensidad del dolor y el consumo analgésico(5).

Así mismo, el manejo del dolor durante la pandemia COVID-19 ha demostrado ser un desafío, en el personal de salud y adaptar rápidamente la telemedicina para un manejo eficaz del dolor(6).

Es necesario realizar una programación de ejercicio físico de 4 días a la semana, durante al menos 50 minutos y con intensidades del 77% de FC max de ejercicio aeróbico o de fuerza en un paciente con dolor crónico, como estrategia para la reducción del dolor. Por otro lado, es necesario tener en consideración que el ejercicio físico puede ser realizado en sus domicilios como mecanismo del control del dolor, durante este periodo de confinamiento social(7). La participación del Tecnólogo médico en terapia física y rehabilitación como parte del equipo multidisciplinario es sumamente importante junto con todo el equipo multidisciplinario en la recuperación del paciente en sus diferentes etapas de su rehabilitación.

La elección de los parámetros con respecto a los ejercicios se debe enfatizar los ejercicios globales en las condiciones de dolor nociplástico, por otro lado, los ejercicios específicos en las condiciones de dolor no nociplástico estarán basados en las condiciones del paciente y las habilidades del personal de terapia física y rehabilitación(8). Además, es necesario tener en consideración, en este periodo de confinamiento social obligatorio realizar actividad física dentro de su entorno para poder mantener un estilo de vida de saludable del individuo y así tener una calidad de vida óptima.

Por último, es necesario gestionar programas educativos en la promoción de comportamientos saludable en la población a fin de mejorar los niveles de actividad física para la prevención de dolor musculoesquelético en los diferentes sectores.
